# miR-103-3p Regulates the Differentiation and Autophagy of Myoblasts by Targeting MAP4

**DOI:** 10.3390/ijms24044130

**Published:** 2023-02-18

**Authors:** Xianxian Zhang, Shihui Huang, Xi Niu, Sheng Li, Jiafu Wang, Xueqin Ran

**Affiliations:** 1 College of Life Science, Institute of Agro-Bioengineering, Guizhou University, Guiyang 550025, China; 2 College of Animal Science, Key Laboratory of Animal Genetics, Breeding and Reproduction in the Plateau Mountainous Region and Plant Resource Conservation and Germplasm Innovation in Mountainous Region (Ministry of Education), Guizhou University, Guiyang 550025, China

**Keywords:** miR-103-3p, differentiation, autophagy, *MAP4*, skeletal muscle

## Abstract

Skeletal muscle is the most abundant tissue in mammals, and myogenesis and differentiation require a series of regulatory factors such as microRNAs (miRNAs). In this study, we found that miR-103-3p was highly expressed in the skeletal muscle of mice, and the effects of miR-103-3p on skeletal muscle development were explored using myoblast C2C12 cells as a model. The results showed that miR-103-3p could significantly reduce myotube formation and restrain the differentiation of C2C12 cells. Additionally, miR-103-3p obviously prevented the production of autolysosomes and inhibited the autophagy of C2C12 cells. Moreover, bioinformatics prediction and dual-luciferase reporter assays confirmed that miR-103-3p could directly target the microtubule-associated protein 4 (*MAP4*) gene. The effects of *MAP4* on the differentiation and autophagy of myoblasts were then elucidated. *MAP4* promoted both the differentiation and autophagy of C2C12 cells, which was contrary to the role of miR-103-3p. Further research revealed that MAP4 colocalized with LC3 in C2C12 cell cytoplasm, and the immunoprecipitation assay showed that MAP4 interacted with autophagy marker LC3 to regulate the autophagy of C2C12 cells. Overall, these results indicated that miR-103-3p regulated the differentiation and autophagy of myoblasts by targeting *MAP4*. These findings enrich the understanding of the regulatory network of miRNAs involved in the myogenesis of skeletal muscle.

## 1. Introduction

Skeletal muscle, which is a highly plastic, is the most abundant tissue in mammals [1]. Myogenesis is a complex-multistep process requiring intricate regulation, needing a variety of signaling pathways, growth factors, and myogenic regulatory transcription factors to coordinate with each other. Myogenic factor 5 (*Myf5*), myogenin (*MyoG*), myoblast determination (*MyoD*), and myogenic factor 6 (*Myf6*) are members of the myogenic regulatory factor (MRF) family, which comprise the core network that governs the development and differentiation of myoblasts. *Myf5* induces the proliferation of myoblasts [2]. Subsequently, *MyoD* begins to express and regulate several muscle-specific genes, such as *MyHC* and *MyoG*, inducing myoblasts to exit the cell cycle and enter differentiation programs; therefore, *MyHC* and *MyoG* are used as markers of myoblast differentiation [3]. Finally, myofilaments recombine, central nuclei migrate to the cell periphery, and myotubules mature under the control of *Myf6* [4]. The processes of myoblast development and differentiation are highly regulated by many other signaling molecules but are still not fully understood, and the identification of regulatory factors for myoblast differentiation is pivotal to clarifying the mechanism of skeletal muscle homeostasis.

In recent years, many studies have found that non-coding RNAs, such as microRNAs, play an important regulatory role in the growth and regeneration of skeletal muscle. In animals, one third of genes are regulated by miRNAs, which have emerged as essential regulators of skeletal muscle growth and regeneration. Among well-researched miRs, those involved in myogenesis include miR-27a [5], miR-99a-5p [6], miR-499, miR-208b, miR-206 and miR-486 [7], miR-1, and miR-133 [8]. Several muscle-related miRNAs have been detected by functional gain and loss experiments in mouse models [9]. However, the interaction of molecules leading to complex structures still need further exploration. miR-103-3p is located in the intron of *PANK3*, a key regulatory enzyme in the biosynthesis of coenzyme A in mammalian cells. Maintaining sufficient levels of CoA is important to support energy metabolism in cells [10]. PANK3 is widely expressed in various tissues and organs, as is miR-103-3p. It has been verified that miR-103-3p participates in a wide variety of cell functions, such as cell apoptosis, proliferation, differentiation, migration, and autophagy, by targeting different genes [11,12,13]. For example, miR-103-3p attenuates neural stem cell differentiation by targeting the *Nde1* gene [14] and inhibits the expression of *Satb2* to suppress Foxc1-promoted differentiation in MC3T3-E1 cells [15]. However, the role of miR-103-3p in skeletal muscle development remains unclear.

Microtubule-associated protein 4 (*MAP4*) is a member of the microtubule-associated protein (MAP) family and is ubiquitously expressed in non-neuronal cells. It plays an important regulatory role in microtubule stability and cell cycle progression [16]. *MAP4* is required for paraxial microtubule organization in muscle cells, and the absence of *MAP4* impairs myoblast elongation and fusion in skeletal muscle [17]. Myofibers are short and apolar, microtubules are disorganized, and normal anisotropic myofibrils are absent in the absence of *MAP4* [18]. However, its effects on skeletal muscle maintenance and the regulatory mechanism need further exploration. 

In this study, we explored the function of miR-103-3p in the growth and development of skeletal muscle. Our results showed that miR-103-3p could target MAP4 to regulate the differentiation and autophagy of myoblasts.

## 2. Results

### 2.1. Expression of miR-103-3p

**Figure 1:**
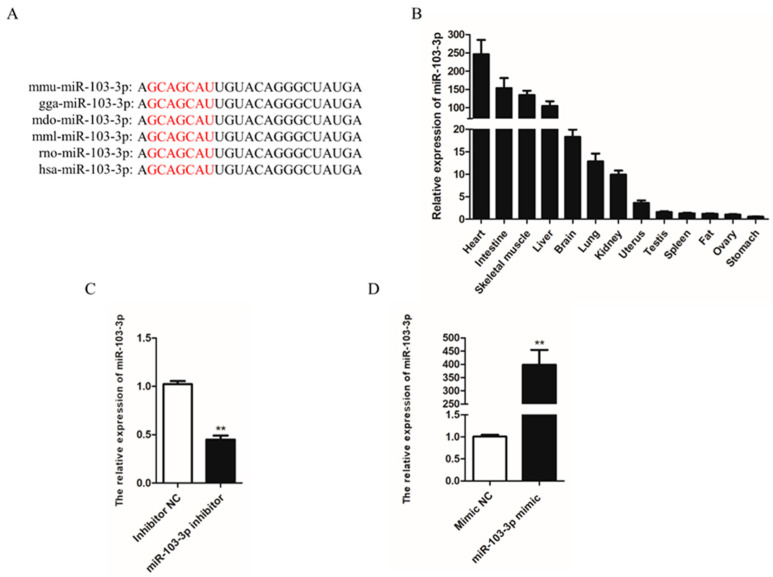
Expression of miR-103-3p in mouse tissues. (**A**) miR-103-3p sequence of different species: mmu, mice; gga, chicken; mdo, *Monodelphis*; mml, *Macaca mulatta*; rno, rat; hsa, *Homo sapiens*. The seed sequence of miR-103-3p is shown in red. (**B**) Expression of miR-103-3p in different mouse tissues. Skeletal muscle was uniformly collected from the gastrocnemius muscle of the right calf from three mice. (**C**,**D**) The expression of miR-103-3p in C2C12 cells was detected by qRT-PCR after transfection with the miR-103-3p inhibitor or mimic. *n* = 3, ** *p* < 0.01.

Sequence analysis indicated that the mature sequences of miR-103-3p were broadly conserved and there was no difference among different species (Figure 1A). To explore the expression pattern of miR-103-3p in mouse tissues, the expression of miR-103-3p was examined by qRT-PCR. The results showed that the expression level of miR-103-3p in skeletal muscle tissue was relatively higher than that in the other tissues, except for the heart and intestine (Figure 1B). Then, murine C2C12 cells were transfected with inhibitors or mimics to knock down or overexpress miR-103-3p in order to identify its potential roles in myoblasts. The expression of miR-103-3p was significantly decreased by transfection with an miR-103-3p inhibitor (Figure 1C) and increased more than 400-fold by transfection with an miR-103-3p mimic in C2C12 cells (Figure 1D), compared with the negative control (NC).

### 2.2. miR-103-3p Inhibited the Differentiation of C2C12 Cells

**Figure 2:**
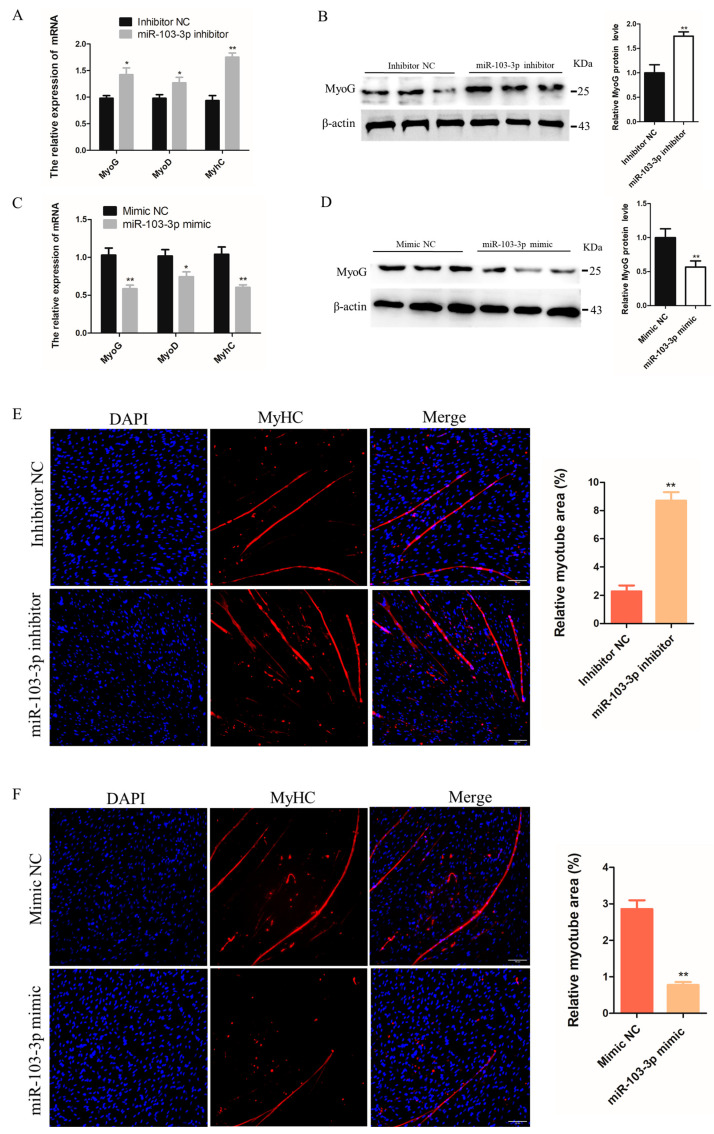
miR-103-3p inhibited the differentiation of C2C12 cells. (**A**,**C**) The mRNA expression of *MyoG*, *MyoD*, and *MyHC* in C2C12 cells was determined by qRT-PCR at 24 h after transfection with the miR-103-3p inhibitor or mimic. *n* = 3. (**B**,**D**) The protein expression of MyoG in C2C12 cells was determined by western blot at 48 h after transfection with the miR-103-3p inhibitor or mimic. β-Actin was used as the endogenous control. *n* = 3 (**E**,**F**) C2C12 cells were induced to differentiate for 48 h. Anti-MyHC immunofluorescence staining was performed in C2C12 cells at 48 h after transfection with the miR-103-3p mimic or inhibitor, and the relative myotube area was measured following miR-103-3p knockdown and overexpression. Scale bar = 100 μm. *n* = 3. * *p* < 0.05, ** *p* < 0.01.

To investigate the effects of miR-103-3p on the differentiation of myoblasts, the growth medium was changed to differentiation medium to encourage the differentiation of C2C12 cells when the cell density reached approximately 70–80% confluence, and inhibitors or mimics of miR-103-3p were transfected to knock down and overexpress miR-103-3p in C2C12 cells. The mRNA expression of muscle differentiation marker genes *MyoG*, *MyoD*, and *MyHC* was significantly increased in C2C12 cells after transfection with the miR-103-3p inhibitor (Figure 2A); similarly, transfection with the miR-103-3p inhibitor also significantly increased the protein expression of MyoG (Figure 2B). In contrast, the mRNA expression of *MyoG*, *MyoD*, and *MyHC* and the protein expression of MyoG were significantly decreased in C2C12 cells transfected with the miR-103-3p mimic (Figure 2C,D). Myoblast differentiation phenotypes were assessed by the efficiency of cells to fuse and form myotubes [19]. The myotube relative area was significantly improved in C2C12 cells with miR-103-3p knockdown (Figure 2E), while it was decreased with miR-103-3p overexpression, as determined by immunofluorescence staining for MyHC (Figure 2F). These results suggested that miR-103-3p negatively regulated C2C12 cell differentiation.

### 2.3. miR-103-3p Inhibited Autophagy of C2C12 Cells

**Figure 3:**
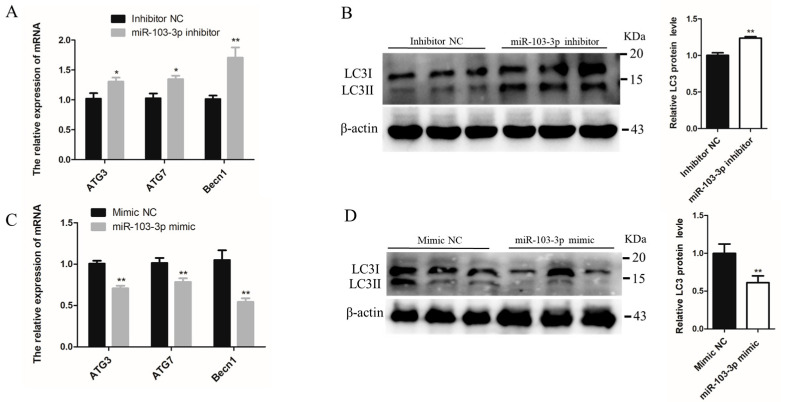

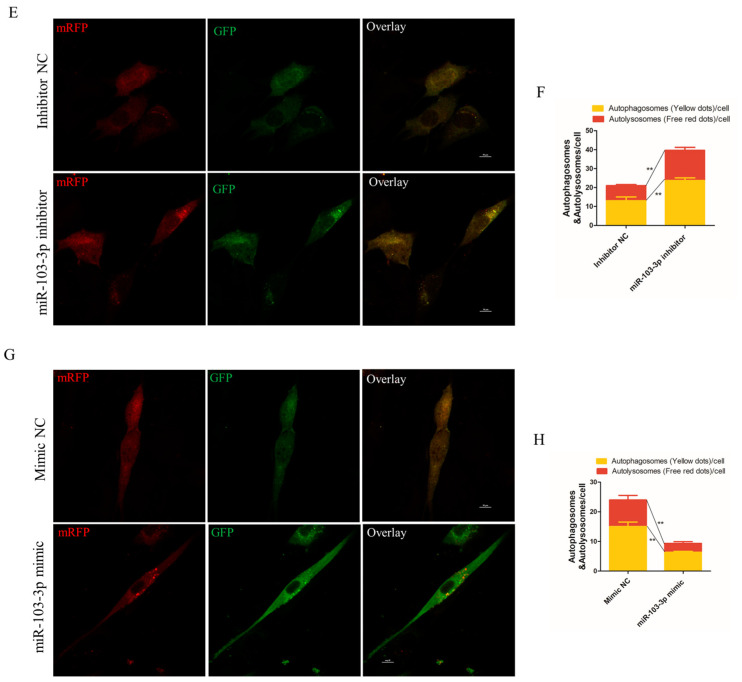
miR-103-3p inhibited autophagy of C2C12 cells. (**A**,**C**) The mRNA expression of *ATG3*, *ATG7*, and *Becn1* in C2C12 cells was determined by qRT-PCR at 24 h after transfection with the miR-103-3p inhibitor or mimic. *n* = 3. (**B**,**D**) The protein expression of LC3 in C2C12 cells was determined by western blot at 48 h after transfection with the miR-103-3p inhibitor or mimic. β-Actin was used as the endogenous control. *n* = 3. (**E**,**G**) The autophagosomes and autolysosomes of C2C12 cells with miR-103-3p knockdown or overexpression were visualized by confocal microscopy at 24 h after treatment with the mRFP-GFP-LC3 adenovirus. Red fluorescence indicates autolysosomes; green fluorescence indicates lysosomes; yellow fluorescence indicates autophagosomes. Scale bar = 10 μm. (**F**,**H**) Mean numbers of autophagosomes and autolysosomes in overlay images per cell. *n* = 3. * *p* < 0.05, ** *p* < 0.01.

The role of miR-103-3p in autophagy was determined by miR-103-3p knockdown and overexpression in C2C12 cells. When miR-103-3p was silenced, the mRNA levels of autophagy-related genes, such as autophagy-related 3 (*ATG3*), autophagy-related 7 (*ATG7*) and Beclin1 (*Becn1*), were significantly enhanced in C2C12 cells (Figure 3A). Microtubule-associated protein light chain 3 (*LC3*) is widely used to evaluate autophagy, and the conversion of LC3 I localized in the cytoplasm to LC3 II located on the autophagosome membrane is a hallmark of autophagy [20]. Our results indicated that miR-103-3p knockdown significantly enhanced the protein levels of LC3 (Figure 3B). In contrast, miR-103-3p overexpression reduced the mRNA expression of *ATG3*, *ATG7*, and *Becn1* and the protein expression of LC3 (Figure 3C,D). In addition, autophagic flux was evaluated using the mRFP-GFP-LC3 reporter. It was observed that the GFP fluorescence of the tandem autophagosome marker was lost upon autophagosome fusion with lysosomes. In the C2C12 cells with miR-103-3p knockdown, the mRFP-GFP-LC3 reporter showed that levels of autophagosome and autolysosome formation were both increased (Figure 3E,F), but the level was decreased in C2C12 cells with miR-103-3p overexpression (Figure 3G,H). These results provided support that miR-103-3p could negatively regulate the autophagy of C2C12 cells.

### 2.4. miR-103-3p Suppressed MAP4 Expression

**Figure 4:**
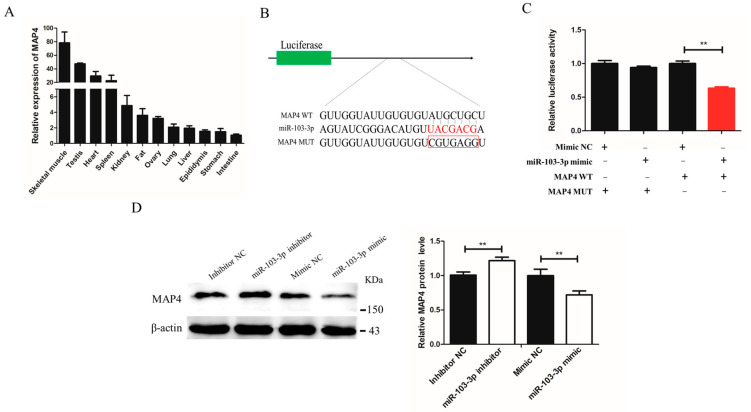
miR-103-3p directly targeted the *MAP4* gene. (**A**) Expression of *MAP4* in different mouse tissues. *n* = 3. (**B**) The miR-103-3p binding site in *MAP4* 3′-UTR with the seed sequence of miR-103-3p and the mutant sequence highlighted in red and in the red box, respectively. (**C**) The relative luciferase activity was analyzed in HEK293t cells that were co-transfected with wild-type or mutant *MAP4* 3′-UTR with the miR-103-3p mimic or mimic NC by the dual-luciferase reporter assay. The relative luciferase activity was assayed at 48 h. *n* = 3. (**D**) Western blot analysis of the protein level of *MAP4* in C2C12 cells at 48 h after transfection with miR-103-3p inhibitor or mimic. β-Actin was used as endogenous control. Data are expressed as the mean ± SEM, *n* = 3. ** *p* < 0.01.

To further investigate the mechanism underlying miR-103-3p inhibition of differentiation and autophagy of myoblasts, TargetScan (https://www.targetscan.org/vert_72/; accessed on 1 November 2021) was used to identify differentiation- and autophagy-related genes containing miR-103-3p response elements in their 3′-UTRs. It was predicted that *MAP4* might be the candidate target gene of miR-103-3p. First, the expression level of *MAP4* was determined to be the highest in mouse tissues (Figure 4A). Then, bioinformatics analysis was performed, revealing that seven bases on the *MAP4* 3′-UTR were complementary to the seed sequences of miR-103-3p. A dual-luciferase reporter assay was further performed to confirm the presence of miR-103-3p-responsive components in the *MAP4* 3′-UTR (Figure 4B). The results indicated that miR-103-3p could combine with the site of the wild-type reporter, but not the mutant-type reporter of the *MAP4* 3′-UTR (Figure 4C). Moreover, the protein expression of *MAP4* was blocked after transfection with the miR-103-3p mimic, but transfection with the miR-103-3p inhibitor could promote the expression of *MAP4* (Figure 4D). Collectively, these results proved that miR-103-3p directly targeted the *MAP4* gene to control *MAP4* expression in C2C12 cells.

### 2.5. MAP4 Promoted the Differentiation of C2C12 Cells

**Figure 5:**
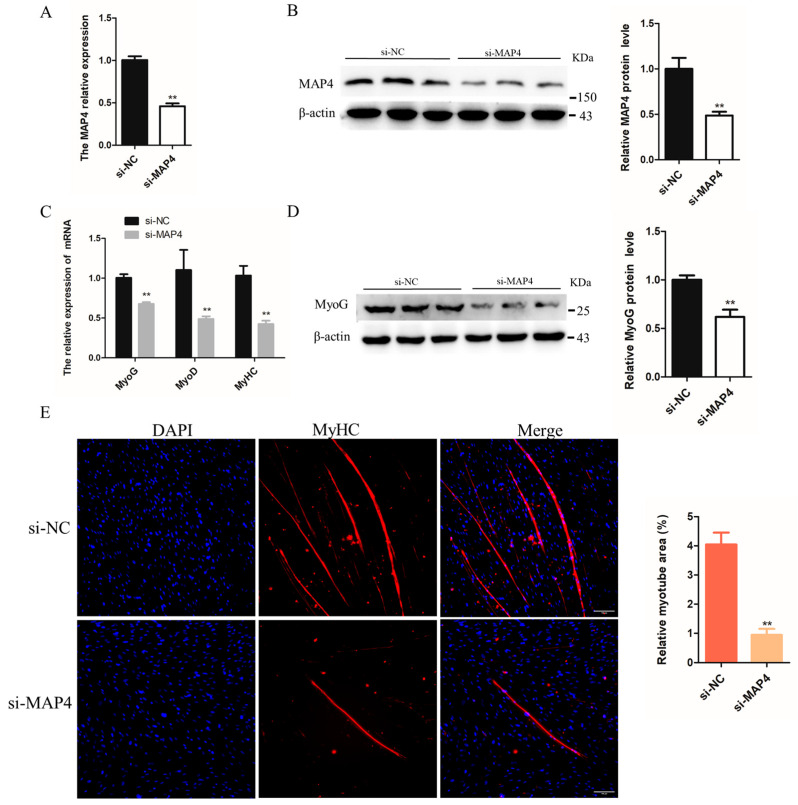

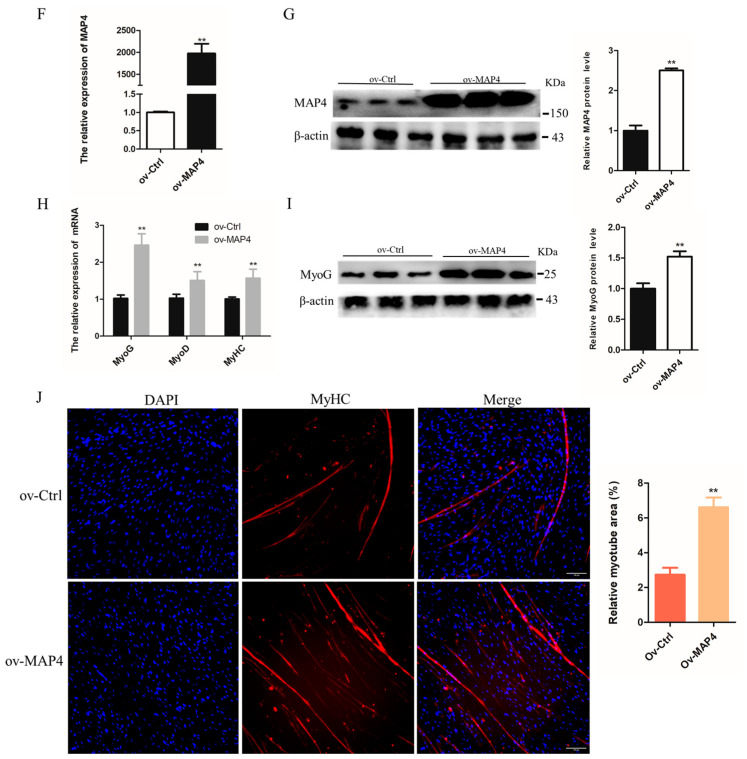
*MAP4* promoted the differentiation of C2C12 cells. (**A**,**C**,**F**,**H**) The mRNA expression of *MAP4*, *MyoG*, *MyoD*, and *MyHC* in C2C12 cells was detected by qRT-PCR at 24 h after transfection with *MAP4* siRNA or MAP4-overexpressing plasmids. *n* = 3. (**B**,**D**,**G**,**I**) The protein expression of MAP4 in C2C12 cells was detected by western blot at 48 h after transfection with *MAP4* siRNA or *MAP4*-overexpressing plasmids. β-Actin was used as the endogenous control. *n* = 3. (**E**,**J**) Anti-MyHC immunofluorescence staining was performed in C2C12 cells at 48 h after transfection with *MAP4* siRNA or *MAP4*-overexpressing plasmids, and the relative myotube area was measured following *MAP4* knockdown and overexpression. Scale bar = 100 μm. *n* = 3. ** *p* < 0.01.

To determine the role of *MAP4* in myoblast differentiation, *MAP4* siRNA was transfected into C2C12 cells to reduce the *MAP4* mRNA and protein expression levels (Figure 5A,B). After *MAP4* knockdown, the mRNA levels of muscle differentiation-related genes *MyoG*, *MyoD*, and *MyHC* and protein expression of MyoG were significantly inhibited (Figure 5C,D). Moreover, the relative myotube area was significantly reduced and myotube formation was restrained after *MAP4* knockdown, based on the MyHC immunofluorescence assay (Figure 5E). *MAP4* knockdown restrained the differentiation of C2C12 cells.

The effects of *MAP4* overexpression on the differentiation of C2C12 cells were also examined. After inducing overexpression of *MAP4* by transfection with the *MAP4*-overexpressing plasmid, the expression level of *MAP4* was significantly increased (Figure 5F,G). The mRNA levels of *MyoG*, *MyoD*, and *MyHC* and protein levels of MyoG were significantly increased in C2C12 cells after *MAP4* overexpression (Figure 5H,I). Additionally, the relative myotube area was significantly enhanced and myotube formation was promoted in C2C12 cells after *MAP4* overexpression (Figure 5J). *MAP4* overexpression promoted the differentiation of C2C12 cells. Collectively, the experiments suggested that *MAP4* had a positive effect on the differentiation of myoblasts. 

### 2.6. MAP4 Induced Autophagy of C2C12 Cells

**Figure 6:**
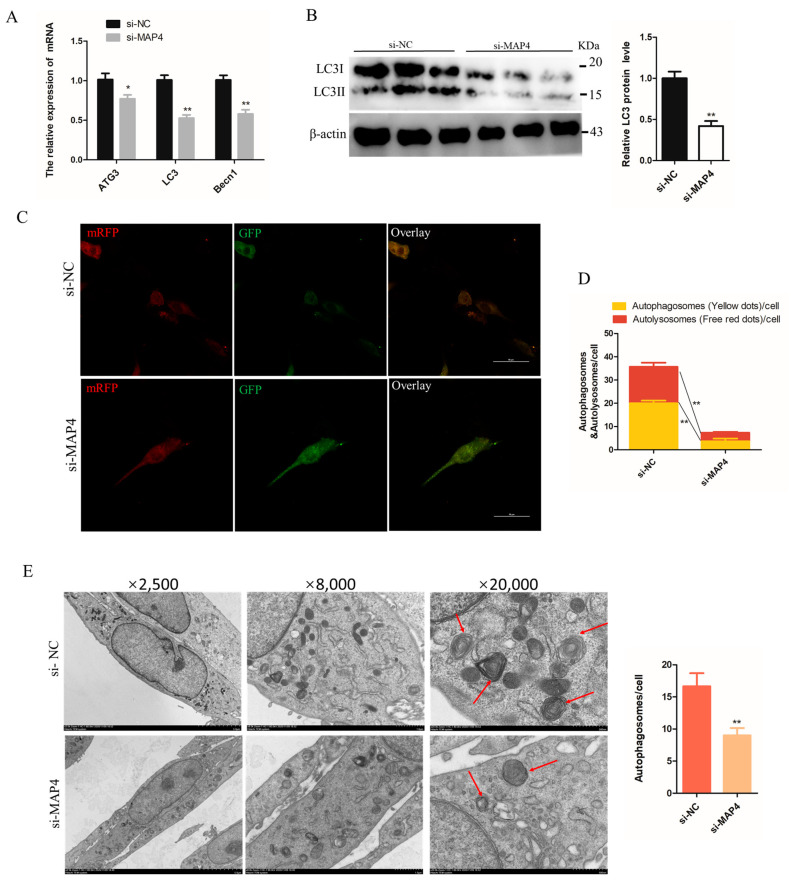

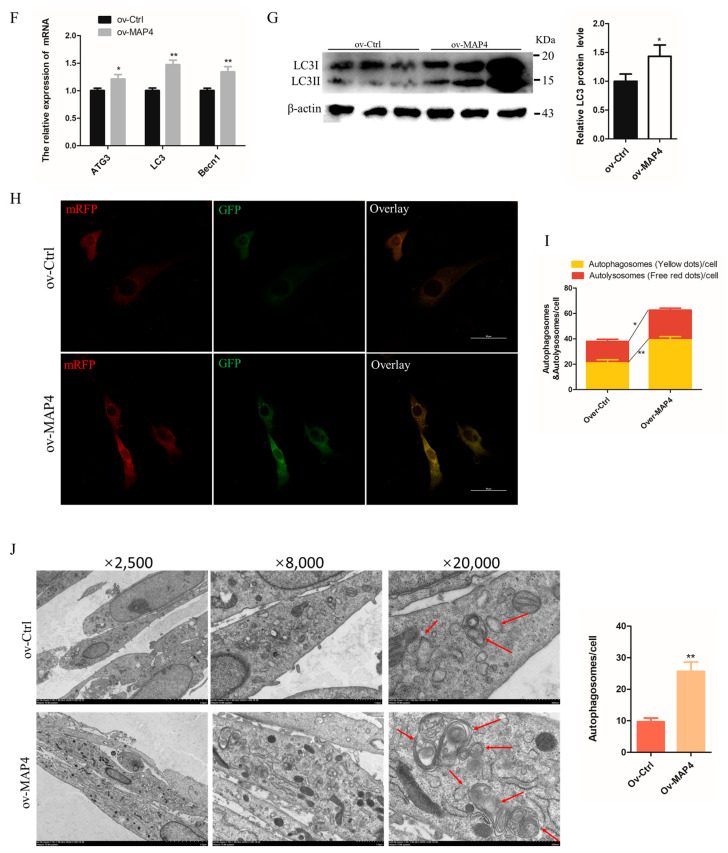
*MAP4* induced autophagy of C2C12 cells. (**A**,**F**) *ATG3*, *LC3*, and *Becn1* expression levels in C2C12 cells were determined at 24 h after *MAP4* knockdown or overexpression. *n* = 3. (**B**,**G**) The protein expression of LC3 was determined by western blot at 48 h after MAP4 knockdown or overexpression. β-Actin was used as endogenous control. *n* = 3. (**C**,**H**) The autophagosomes and autolysosomes in C2C12 cells with *MAP4* knockdown or overexpression were observed by confocal microscopy at 24 h after treatment with the mRFP-GFP-LC3B adenovirus. Red fluorescence indicates autolysosomes; green fluorescence indicates lysosome; yellow fluorescence indicates autophagosomes. Scale bar = 50 μm. *n* = 3. (**D**,**I**) Mean numbers of autophagosomes and autolysosomes in overlay images per cell. (**E**,**J**) Autophagosomes were observed by transmission electron microscopy at 24 h after *MAP4* knockdown or overexpression. Red arrows indicate autophagosomes. Magnification: ×2500 (scale bar = 5 μm), ×8000 (scale bar = 1 μm), and ×20,000 (scale bar = 500 nm). *n* = 3. * *p* < 0.05, ** *p* < 0.01.

The effects of *MAP4* on autophagy were investigated in C2C12 cells. Under the condition of *MAP4* interference, the mRNA levels of the autophagy-related genes (*ATG3*, *LC3* and *Becn1*) and protein expression of LC3 were significantly decreased (Figure 6A,B). Moreover, a stable tandem mRFP-GFP-LC3 was constructed to evaluate autophagic flux in cultured C2C12 cells. The green fluorescence was hardly quenched (Figure 6C) and the numbers of autophagosomes and autolysosomes both significantly declined in *MAP4*-silenced cells (Figure 6D), which implied that autolysosome formation was blocked. The transmission electron microscope images also provided evidence that the number of autophagosomes decreased after *MAP4* knockdown (Figure 6E). These results suggested that *MAP4* knockdown prevented the autophagy of C2C12 cells.

On the other hand, the mRNA expression of *ATG3*, *LC3*, and *Becn1* (Figure 6F) and protein levels of LC3 were significantly increased in *MAP4*-overexpressing cells (Figure 6G). The tandem mRFP-GFP-LC3 system showed that green fluorescence was significantly reduced with enhanced expression of *MAP4* (Figure 6H) and the numbers of autophagosomes and autolysosome were both significantly increased in *MAP4*-overexpressing cells (Figure 6I). Additionally, many larger autophagosomes and autolysosomes could be observed in *MAP4*-overexpressing cells by transmission electron microscopy (Figure 6J), suggesting that *MAP4* overexpression promoted the production of autophagosomes and fusion of autolysosomes. These findings indicated that *MAP4* could induce the autophagy of C2C12 cells.

### 2.7. MAP4 Interacted with LC3

**Figure 7:**
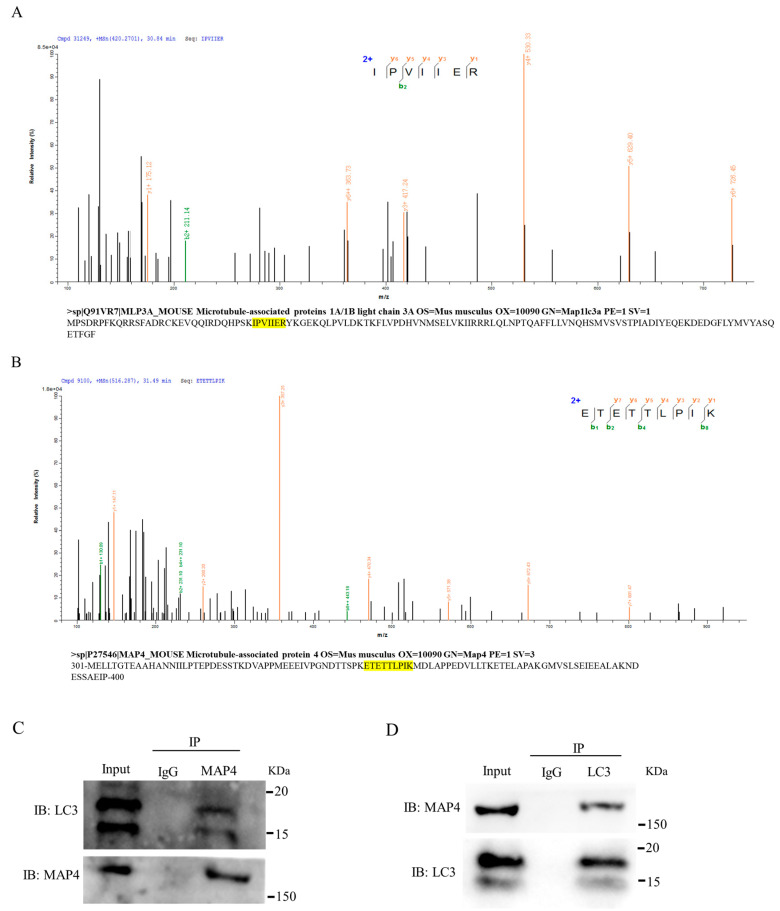

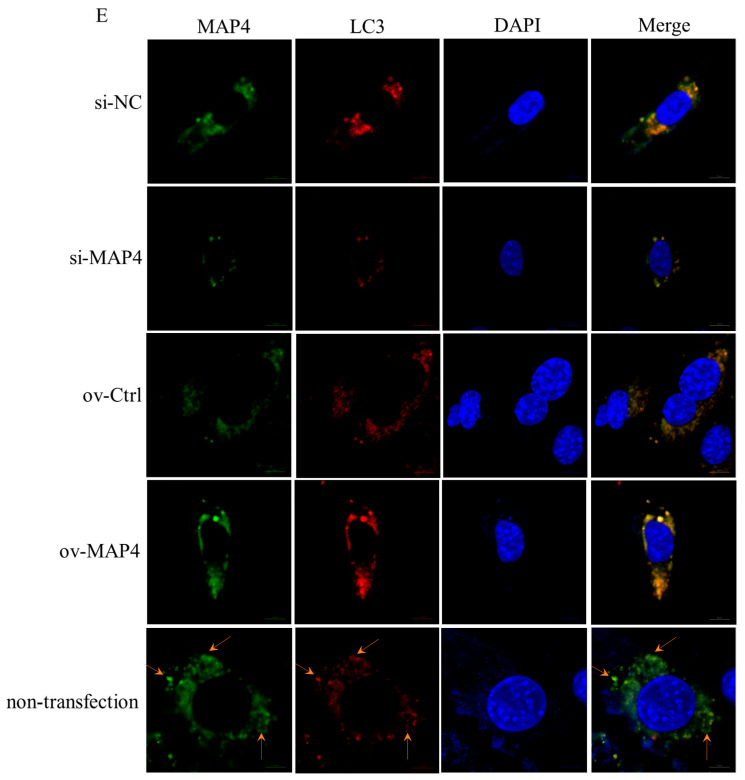
MAP4 interacted with LC3. (**A**) MS/MS spectra of the peptide (IPVIIER) assigned to LC3 from MAP4 immunoprecipitates and the binding peptide within the LC3 amino acid sequence. The yellow highlights is amino acid sequence of LC3. (**B**) MS/MS spectra of the peptide (ETETTLPIK) assigned to MAP4 from LC3 immunoprecipitates and the location within the MAP4 amino acid sequence. The yellow highlights is amino acid sequence of MAP4. Amino acid sequences of LC3 and MAP4 were obtained from Uniprot (https://www.uniprot.org/; accessed on 1 May 2022). (**C**) The immunoprecipitates of MAP4 were purified using anti-MAP4, and the presence of LC3 and MAP4 was analyzed by western blotting. (**D**) The immunoprecipitates of LC3 were purified using anti-LC3, and the presence of MAP4 and LC3 was analyzed by western blotting. (**E**) Immunofluorescence of LC3 (red) and MAP4 (green) was imaged using a confocal microscope, the cell nuclei were visualized by DAPI staining (blue). Yellow arrows indicate the co-localization of MAP4 and LC3. Scale bar = 10 μm.

To reveal the mechanism underlying *MAP4* regulation of autophagy of C2C12 cells, co-immunoprecipitation assays coupled with mass spectrometry (CoIP-MS) were used to screen for proteins that interacted with MAP4. The autophagy marker LC3 was identified from the MAP4 immunoprecipitates (Figure 7A) and vice versa; MAP4 was screened from the LC3 immunoprecipitates (Figure 7B). Co-immunoprecipitation combined with western blotting further verified the interactions between MAP4 and LC3 proteins (Figure 7C,D). In addition, the distribution of MAP4 and LC3 proteins in myoblasts was similar in C2C12 cells, as determined by immunofluorescence staining, indicating that endogenous MAP4 colocalized with LC3 in C2C12 cell cytoplasm (Figure 7E). Additionally, the expression of LC3 varied along with increasing or decreasing MAP4 expression (Figure 7E and Figure 6B,G, respectively). These results robustly presented direct interactions between MAP4 and LC3 in the regulation of autophagy of C2C12 cells.

## 3. Discussion

In recent years, many studies have revealed that miRNAs are essential regulators of skeletal muscle growth and development. The loss of miR-103-3p expression induced autophagy by directly targeting *Atg5* in hypoxia-induced H9c2 cardiomyocytes [21] and targeting *SOX2* in LPS-injured PC12 cells [13]. In our study, the expression levels of miR-103-3p in skeletal muscle were relatively higher among the tissues examined, so we speculated that the high levels of miR-103-3p in skeletal muscle might have a function in myocytes. Our results showed that miR-103-3p negatively regulated the autophagy of C2C12 cells. Becn1 is a core component of the autophagy-promoting PtdIns3K complex involved in autophagic vesicle nucleation [22]. LC3 is a structural protein of the autophagosome; its function is mainly related to the formation of autophagosomes. The LC3 precursor is processed by ATG4 to form the cytoplasmic LC3-I, which is activated by AGT7 and transferred to ATG3 to form the membrane-bound LC3-II, which can be attached to the membrane of the autophagosome [23,24]. We found that the expression levels of *Becn1*, *ATG3*, *ATG7*, and *LC3* gradually decreased with increasing miR-103-3p expression and autophagic flux was reduced with increased miR-103-3p expression in C2C12 cells. Therefore, the high expression of miR-103-3p may lead to autophagic deficiency by inhibiting the expression of *Becn1*, *ATG3*, *ATG7*, and *LC3*.

In addition, miR-103-3p inhibited myoblast differentiation by decreasing the expression levels of muscle differentiation marker genes (*MyoD*, *MyoG*, and *MyHC*), and the efficiency of C2C12 cells to fuse and form myotubes was impaired when miR-103-3p was overexpressed. Many studies have proven that autophagy is necessary for myogenic differentiation. Autophagic signals are further activated during myoblast differentiation and upregulated in the whole differentiation process of myoblasts, both in vivo and in vitro [25,26,27,28]. Disruption of autophagy hinders myofiber formation whether at the initiation, cargo trafficking, or autolysosome fusion steps [25,26,29]. Our results established miR-103-3p as a critical modulator that coordinates both the differentiation and autophagy of myoblasts, adding miR-103-3p to the growing list of components in the regulation of skeletal muscle development.

miRNAs perform biological functions via targeting different genes, resulting in translational repression and mRNA decay [30], and many studies have shown that one miRNA can regulate multiple target genes, which is also true for miR-103-3p. Specifically, previous reports indicated that miR-103-3p participated in different biological processes by targeting different genes, such as *Nde1* [14], *Satb2* [15], *ATG5* [21], *CCNE1*, and *TFDP2* [11]. In this study, bioinformatic analysis and dual-luciferase reporter assays confirmed that *MAP4* was a target gene of miR-103-3p in C2C12 cells. Moreover, the protein expression of MAP4 exhibited active responses upon miR-103-3p increase or decrease. Thus, we confirmed that miR-103-3p could directly regulate *MAP4* gene expression in C2C12 cells. As for whether miR-103-3p targets other genes during the differentiation and autophagy of myoblasts, more research is needed.

The expression and function of *MAP4* in the myoblasts of muscle were then investigated. MAP4 belongs to the family of MAPs, which are crucial cytosolic skeletal proteins and widely expressed [31]. Our findings were consistent in that *MAP4* was widely expressed in all examined tissues. Previous research indicated that *MAP4* is essential for myogenesis as a microtubule organizing factor since depletion of *MAP4* impairs muscle cell differentiation and elongation [17]. Additionally, *MAP4* is vital for myotube formation since fusion occurred in C2C12 cells in the absence of *MAP4*; the myotubes were short and apolar, the microtubules were disorganized, and normal anisotropic myofibrils were absent [18]. In our study, *MAP4* knockdown significantly inhibited myotube formation, while *MAP4* overexpression promoted myotube formation by upregulation of marker genes (*MyoD*, *MyoG* and *MyHC*) for myoblast differentiation. These results validated that *MAP4* plays a positive role in C2C12 cell differentiation, which was regulated by miR-103-3p. 

MAP4 is also located in mitochondria, and it is a key mediator in regulating microtubules (MTs) and mitochondria [32]. *MAP4* phosphorylation increases its accumulation in mitochondria, induces mitochondrial apoptosis, and damages microtubule stability [33]. One of the functions of basic autophagy is to eliminate damaged mitochondria in a timely manner and maintain cellular homeostasis [34]. However, the effects of *MAP4* on autophagy remain unclear. Here, we provided clear evidence that MAP4 had a positive role in inducing autophagic flux and autophagosome production by affecting the expression of marker genes (*ATG3*, *Becn1*, and *LC3*) for autophagy. Further experiments revealed that MAP4 colocalized with LC3 in C2C12 cell cytoplasm, and the interaction between MAP4 and LC3 was confirmed by co-immunoprecipitation and CoIP-MS assays. LC3 is widely used to monitor autophagy; the precursor of LC3 is processed into the cytosolic form of LC3-I, and the latter is activated and modified by Atg7 and Atg3 to the membrane-bound form of LC3-II [35]. LC3-II is localized to pre-autophagosomes and autophagosomes, making this protein a useful marker of autophagy [36]. In the present study, we report for the first time that MAP4 interacted with LC3 to regulate the autophagy of C2C12 cells. Autophagy is regulated by microtubules, which promote the formation of autophagosomes [37]; therefore, microtubule-associated proteins, such as LC3 and MAP4, play an important role in autophagy. In this study, we not only found that MAP4 colocalized with LC3 in C2C12 cell cytoplasm, but we also detected that the expression of LC3 was positively regulated by MAP4 together with the numbers of autophagosomes and autolysosomes. This finding suggests that the expression of LC3 precursor or the process of LC3I/II might be indirectly regulated by MAP4, and MAP4 really has a role in the formation of autophagosomes and autolysosomes. We hypothesize that *MAP4* plays a key role in autophagosome maturation and autophagosome membrane extension through interaction with LC3. However, the specific regulatory mechanism of MAP4 in autophagy formation needs further exploration. 

**Figure 8:**
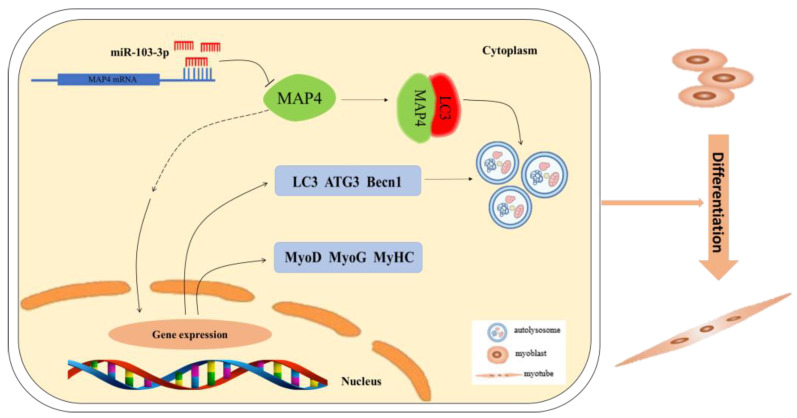
Schematic diagram of the miR-103-3p/MAP4 axis mediating myoblast differentiation and autophagy. miR-103-3p targets MAP4 3′UTR to regulate MAP4 expression, thus mediating differentiation and autophagy of myoblasts.

In summary, our study reveals a new mechanism by which miR-103-3p regulates skeletal muscle development. It was established that miR-103-3p regulated myogenesis by inhibiting the differentiation and autophagy of C2C12 cells via targeting the *MAP4* gene. In addition, MAP4 induced autophagy by interacting with LC3 and differentiation of myoblast cells (Figure 8). The physiological effects of miR-103-3p and *MAP4* are worth further study in vivo.

## 4. Materials and Methods

### 4.1. Cell Culture and Transfection

Cell lines, including mice C2C12 and human HEK293T, were purchased from the American Type Culture Collection (ATCC, Manassas, VA, USA). C2C12 and HEK293T cells were cultured in growth DMEM (Gibco, Grand Island, NY, USA) including 10% fetal bovine serum (FBS) (Gibco, Grand Island, USA) and 1% penicillin-streptomycin (Beyotime, Shanghai, China). When the cell density reached approximately 70–80% confluence, the growth medium was changed to the differentiation medium (DM: DMEM + 2% horse serum (Solarbio, Beijing, China) to encourage C2C12 cell differentiation. All cells were cultured at 37 °C and under a 5% CO_2_ atmosphere. 

miR-103-3p inhibitor (5′-UCAUAGCCCUGUACAAUGCUGCU-3′), inhibitor NC (5′-CAGUACUUUUGUGUAGUACAA-3′), miR-103-3p mimic (5′-AGCAGCAUUGUACAGGGCUAUGA-3′), mimic NC (5′-UUGUACUACACAAAAGUACUG-3′), MAP4 small interfering RNAs (si MAP4) (sense strands: 5′-GAGGAGAUGUCAAGAUUGATT-3′, anti-sense strands: 5′-UCAAUCUUGACAUCUCCUCTT-3′), and siRNA negative control (si NC) (sense strands: 5′-UUCUCCGAACGUGUCACGUTT-3′, anti-sense strands: 5′-ACGUGACACGUUCGGAGAATT-3′) were synthesized by GenePharma (GenePharma, Shanghai, China). The *MAP4* overexpression vector (ov-*MAP4*) was constructed using the pEX-3 vector (GenePharma, Shanghai, China). The dual-luciferase reporter vector was constructed using the pmirGLO dual-luciferase reporter vector (Tsingke Biontech, Beijing, China). The sequence of the overexpressed *MAP4* CD and *MAP4* 3′-UTR (wild and mutant) was obtained from the NCBI and synthesized by Tsingke (Tsingke Biontech, Beijing, China). The CDS sequence of *MAP4* was cloned into the pEX-3 vector and the 3′-UTR of *MAP4* was cloned into the pmirGLO dual-luciferase reporter vector. For transfection, the cells were seeded into 6-well plates with 1 × 10^6^ cells/well. When the cells were grown to approximately 70–80% confluence, the growth medium was changed to the differentiation medium to study cell differentiation. Lipofectamine 3000 reagent (Invitrogen, Carlsbad, CA, USA) was used for cell transfection according to the manufacturer’s instructions. Subsequently, 4 μL Lipofectamine 3000 and 100 pmol oligonucleotides or 5 μg plasmid (with 10 μL P3000 Reagent) were each diluted with 250 μL optimMEM medium. Then, the diluted oligonucleotides or plasmids were added to each tube of diluted Lipofectamine 3000 Reagent (1:1 ratio) and incubated at room temperature for 15 min. The mixture was added to the cell culture plate and mixed well. The transfection efficiency was measured at 24 to 72 h after transfection. 

### 4.2. Quantitative Real-Time PCR (qPCR)

Total RNAs were isolated using Trizol (Solarbio, Beijing, China) and underwent reverse transcription to cDNA using the Promega PrimeScript RT reagent kit (Promega, Madison, WI, USA). Reverse transcription of mRNA and miRNA was performed as previously described [38]. qPCR analysis was performed on the synthesized cDNA using Talent qPCR PreMix (SYBR Green) (Tiangen, Beijing, China) with the appropriate amplification conditions: 95 °C, 3 min; 95 °C, 5 s; 50–60 °C, 10 s; 72 °C, 15 s; for 40 cycles. The qPCR primers (Table 1) were designed using Primer Premier 5 (Premier Biosoft, CA, USA) and synthesized by Sangon Biotech (Shanghai, China). The 2^−ΔΔCt^ method was used to quantify the expression levels and the results were normalized relative to β-actin (for mRNA) and U6 (for miRNA). Each sample was repeated three times.

### 4.3. Western Blot and Immunoprecipitation

The total proteins from C2C12 cells were isolated using RIPA buffer (Beyotime, Shanghai, China) and the protein concentration was measured using the BCA protein assay kit (Beyotime, Shanghai, China). The target proteins were separated using SDS-PAGE electrophoresis and transferred to polyvinylidene fluoride (PVDF) membranes. Then, the PVDF membranes were incubated with specific primary antibodies overnight at 4 °C. Excess primary antibody on the PVDF membranes was washed away. Subsequently, the membranes were incubated with secondary antibody for 2 h at 37 °C. Finally, the protein bands were visualized using the enhanced chemiluminescence (ECL) kit (Beyotime, Shanghai, China) and detected using the Tanon-4600 (Tanon, Shanghai, China). Protein band intensities were quantified by Image J software and normalized against β-actin. The following primary antibodies were used: anti-MyoG (orb6492, Birobyt, Cambridge, UK; 1:200), anti-LC3 (83506, Cell Signaling Technology, Boston, MA, USA; 1:1000), anti-MAP4 (ab245578, Abcam, London, UK; 1:2000), and anti-β-actin (AF7018, Affinity, Changzhou, China; 1:5000). The immunoprecipitation analysis was performed as previously described [39]. Briefly, C2C12 cells were isolated with RIPA buffer and total proteins were immunoprecipitated with protein G beads. Western blotting was used to analyze the immunoprecipitated products.

### 4.4. Dual-Luciferase Reporter Assay

HEK293T cells were used for the dual-luciferase reporter assay. HEK293T cells were incubated in growth medium with 10% FBS (Gibco, Grand Island, USA) and 1% penicillin-streptomycin (Solarbio, Beijing, China) at 37 °C, under 5% CO_2_, and with saturating humidity. HEK293T cells were seeded into a 48-well plate with 1 × 10^5^ cells/well and incubated in a 37 °C incubator until the cells reached approximately 70–80% confluence. MAP4-WT or MAP4-MUT Dual-Luciferase reporter vector (250 ng) and miR-103-3p mimic (10 pmol) or mimic NC (10 pmol) were co-transfected into HEK293T cells using Lipofectamine 3000. The Dual-luciferase reporter assay kit (Promega, Madison, USA) was used to measure the luminescence values of firefly and Renilla luciferase at 48 h after transfection.

### 4.5. LC3-RFP-GFP Reporter Construct and Confocal Microscopy

The tandem autophagosome reporter of mRFP-GFP-LC3 was used to evaluate the autophagic flux. GFP fluorescence is sensitive to lysosomal proteolysis and quenching in an acidic environment, hence the green fluorescence was weakened upon autophagosome fusion with lysosomes. mRFP-GFP-LC3B adenovirus was constructed by Hanbio (Hanbio, Shanghai, China). C2C12 cells were seeded into 24-well cell culture plates and cultured to 70% fusion. The adenovirus (MOI = 30) were transfected into C2C12 cells. Transfected C2C12 cells were photographed using a confocal microscope (Olympus, Tokyo, Japan) at 40× magnification at 24 h after transfection. The numbers of autophagosomes (yellow dots: merge of GFP and RFP signals) and autolysosomes (free red dots: RFP signal alone) were counted in 5 randomly selected fields. 

### 4.6. Immunofluorescence

C2C12 cells were plated on glass coverslips after transfection for 48 h, washed three times with PBS, and fixed in 4% paraformaldehyde for 10 min. Then, the cells were washed three times with PBS and permeabilized with 0.5% Triton X-100 for 5 min (Solarbio, Beijing, China). After blocking with BSA, the cells were incubated overnight with primary antibodies anti-MyHC (DF9647, Affinity, Changzhou, China), anti-LC3 (Cell Signaling Technology, Boston, USA) and anti-MAP4 (Abcam, London, UK) at 4 °C overnight. The primary antibody was washed out of the cells, which were then incubated with fluorescent secondary antibodies Alexa Fluor 488 anti-rabbit IgG, Alexa Fluor 555 anti-mouse IgG, and Alexa Fluor 555 anti-rabbit IgG (all Beyotime, Beijing, China) in the dark for 1 h at room temperature. The cell nuclei were stained with DAPI (Beyotime, Beijing, China, 1:50) for 5 min. The fluorescence intensity was examined using a confocal microscope (Olympus, Tokyo, Japan) at 200× magnification. Three randomly selected images were taken from each well and ImageJ2 software was first used to quantify the fluorescence intensity. The images were reversed in black and white using the Invert tool, then the Threshold tool was used to adjust the threshold so that only the objects to be measured in the image were selected, and finally, the Measure tool was used to detect the fluorescence intensity.

### 4.7. Transmission Electron Microscopy (TEM)

C2C12 cells were detached from the plates using a manual scraper. After washing with PBS, the cells were fixed in 2% glutaraldehyde for 2 h at ambient temperature, rinsed in pure water three times, and exposed to 1% uranyl acetate for 15 min. The cells were embedded in 1% agarose solution. Sections (80 nm) were cut using a Leica EM UC7 ultramicrotome (Leica, Wetzlar, Germany). The samples were observed using a Hitachi HT7800 TEM (Hitachi, Tokyo, Japan).

### 4.8. Animal Experiments

KM mice were purchased from SPF (Beijing) Biotechnology Co. Ltd. (Beijing, China). Standard pellet diet and water were provided ad libitum and all animals were maintained on a 12 h light/12 h dark cycle in a temperature-controlled room at 21–23 °C. The 8-week-old mice were euthanized by CO_2_ and various tissues were collected and stored in liquid nitrogen to detect the expression of miR-103-3p and MAP4. All experimental procedures involving animals were approved by the Institutional Animal Care Committee of Guizhou University (No. EAE-GZU-2021-E022; approval date: 12 November 2021), and were carried out in accordance with the National Research Council’s Guide for the Care and Use of Laboratory Animals.

### 4.9. Statistical Analysis

All data were analyzed based on non-parametric tests using IBM SPSS Statistics v.20.0 software (IBM Corp., Armonk, NY, USA). Comparisons of two groups were based on unpaired Student’s *t*-tests and comparisons of more than two groups were based on one-way ANOVA tests. All data were derived from at least three biological replicates and three experimental replicates (*n* = 3). GraphPad 8.0 (GraphPad, San Diego, CA, USA) was used to plot the results. The results are shown as the mean ± SEM. Values of * *p* < 0.05 and ** *p* < 0.01 were considered to indicate statistical significance.

## Figures and Tables

**Table 1:** Primers for qPCR.

Genes	Forward Primer (5′⟶3′)	Reverse Primer (5′⟶3′)
*MAP4*	AGCTTTTGTTTCCAGCCTCA	TCTGAAGGATTGTCTCGCTCTG
*β-actin*	GTGACGTTGACATCCGTAAAGA	GCCGGACTCATCGTACTCC
*MyoG*	GGTGCCCAGTGAATGCAAC	AGATTGTGGGCGTCTGTAGG
*MyoD*	CGCCTGAGCAAAGTGAATGA	GCAGACCTTCGATGTAGCGG
*MyHC*	AAAATGAAGGGGACGCTGGAG	GTGGTGCCAAAATGGATGCG
*LC3*	AGATCCCAGTGATTATAGAGCGA	CATGTTCACGTGGTCAGGCA
*ATG3*	TCCCACCACCTCCTATGTGT	TATGAACACCAAGCTCTCCCC
*ATG7*	CCCCATGCTCCTCAACAAGT	CTTCGGCTCGACACAGATCA
*Becn1*	TGAATGAGGATGACAGTGAGCA	CACCTGGTTCTCCACACTCTTG
miR-103-3p	CAGCAUUGUACAGGGCUAUGA	TCCAGTTTTTTTTTTTTTTTCAAACAC
U6	CTCGCTTCGGCAGCACA	TCCAGTTTTTTTTTTTTTTTCAAACAC

## Data Availability

The data presented in this study.
